# Crosstalk between the microbiome and immune microenvironment in the pathogenesis and treatment of thyroid carcinoma: a narrative review

**DOI:** 10.3389/fimmu.2026.1782596

**Published:** 2026-09-10

**Authors:** Wanzhi Chen, Jichun Yu, Guoxue Fu, Zhenquan Hong, Xinxin Zhang, Meijun Zhong

**Affiliations:** 1 Department of Thyroid Surgery, The Second Affiliated Hospital of Nanchang University, Nanchang, Jiangxi, China; 2 Department of Pathology, The Second Affiliated Hospital of Nanchang University, Nanchang, Jiangxi, China; 3 Department of General Practice, The Second Affiliated Hospital of Nanchang University, Nanchang, Jiangxi, China

**Keywords:** cancer immunotherapy, immune microenvironment, microbiome, progression, review, thyroid carcinoma

## Abstract

Thyroid carcinoma (TC) is the most common cancer of the endocrine system worldwide, and its incidence has remained stable over the past forty years. Complex hormonal and environmental factors influence TC progression. The gut microbiome, a pivotal mediator in maintaining host physiology and the development of pathology, serves as a biomarker and therapeutic target in cancer immunotherapy. The microbiome-assisted tumor microenvironment (TME) sustains tumors through the modulation of multiple mechanisms. Immune suppression in the TME induces a metastatic phenotype of tumor cells by modulating signaling pathways, cell differentiation, and the innate immune response. Current research has confirmed that gut dysbiosis, bacterial outer membrane components (such as lipopolysaccharides and LPS), and metabolites (such as short-chain fatty acids and SCFAs) have bidirectional regulatory effects on thyroid function. However, the role of the local microbiome in modulating treatment responses and its interactions with the immune TME of TC remains unknown. The literature was searched in PubMed, Web of Science, and Google Scholar using keywords associated with “microbiome” and “thyroid carcinoma”. Research articles, clinical trials, letters, and meta-analyses were published by January 2026. This narrative review summarizes the role of the intratumor microbiome in tumor progression, interactions with chemotherapy, radiotherapy, and targeted therapies. The associations between the microbiome and the immune TME of cancers, as well as the characteristics of the immune TME and alterations in the gut/intratumor microbiome in patients with TC receiving different therapies, are discussed. Mediating microbiome and metabolites might be potential strategies for optimizing personalized therapeutic interventions against TC. Future research should focus on defining microbial signatures associated with treatment success and developing targeted strategies to improve patient outcomes on the basis of microbiome modulation.

## 1 Introduction

Thyroid cancer (TC) is among the most prevalent endocrine tumors and head and neck tumors. Its pathological origins can be divided into two main categories: thyroid follicular epithelial cells and parafollicular cells (1). The main subtypes include papillary thyroid carcinoma (PTC, 80–85%), follicular thyroid carcinoma (FTC, 10–15%), medullary thyroid carcinoma (MTC, <2%), and anaplastic thyroid carcinoma (ATC, <2%) (2). Among these, PTC is the most common and has a favorable prognosis. Global epidemiological data has shown that the age-standardized incidence of TC was 10.1 cases per 100,000 females and 3.1 cases per 100,000 males in 2020 (3). Since 1970, the incidence of TC has remained stable. These inconsistencies may result from variations in sample processing and experimental detection methods across studies as well as that each person has a unique microbiome (above 7. Conclusion). The five-year survival rate of TC is about 98% (4, 5). Pathological diagnoses vary among TC subtypes. PTC is usually diagnosed when the patient or clinical examination results in gland enlargement, often accompanied by difficulty swallowing or breathing (6). FTC typically manifests as a single thyroid nodule with thyroid inflammation or nodular hyperplasia, which is challenging to confirm through simple palpation and usually requires pathological examination to reveal follicular cells lacking nuclear heterogeneity. The diagnosis is primarily based on capsular and vascular invasion (7). MTC presents similarly to PTC and FTC as thyroid nodules, but MTC is characterized by excessive calcitonin secretion. Diagnosis typically involves measuring serum calcitonin and carcinoembryonic antigen levels (8). ATC usually presents as a rapidly growing hard mass that has already metastasized at diagnosis and is classified as a stage IV tumor. Therefore, ATC diagnosis requires ultrasound, computed tomography, magnetic resonance imaging, and fine-needle aspiration biopsy to assess tumor extent, local infiltration, and possible distant metastases (9).

Traditional treatments for TC include thyroidectomy and radioactive iodine therapy, which have achieved good outcomes in most TC patients (10). However, for some aggressive FTCs and advanced ATCs with invasive and metastatic characteristics, traditional therapies are less effective (11). Recent advancements in immunotherapy (primarily checkpoint inhibitors) (12) and molecular targeted therapies (13) have shown significant efficacy for highly malignant TC subtypes such as ATC. Conventional treatments for TC are often associated with various side effects. For example, thyroid-stimulating hormone suppression therapy can lead to hypothyroidism. Radioactive iodine therapy is associated with complications such as gastrointestinal symptoms, bone marrow suppression, and radiation thyroiditis in the early stages and with secondary tumors and pulmonary fibrosis in the later stages (14).

The tumor microenvironment (TME) refers to the internal environment of a tumor, including tumor cells, the tumor microbiome (TM), immune cells, surrounding blood vessels, cancer-associated fibroblasts (CAFs), and the extracellular matrix, which play critical roles in the initiation and progression of cancer (15). Among the immune cells in the TME, M2 macrophages (TAMs) and Tregs promote tumor growth by inducing immunosuppression. B cells exhibit both antitumor and protumor effects in cancer. NK cells have potent anticancer effects by eliminating cancer-transforming cells and enhancing T-cell and antibody responses (16). TMs typically originate from the gut microbiome (GM), and also from the oral cavity and adjacent tissues (17). TMs have been confirmed to be involved in the progression of various cancers, such as liver cancer, pancreatic cancer, colorectal cancer, esophageal cancer, breast cancer, lung cancer (18), and uveal melanoma (19). Studies have revealed that the TM can directly intervene in tumor progression. For example, *Akkermansia muciniphila* (Akk) migrates into the bloodstream, colonizes lung cancer tissues, and subsequently inhibits tumorigenesis by influencing the TM and reprogramming tumor metabolism (20). Metabolites produced by the TM can modulate tumor progression by mediating metabolic and immune homeostasis. For instance, the metabolite indole-3-lactic acid produced by *Lactobacillus* L168 affects dendritic cells (DCs), promoting the production of IL-12 to activate CD8+ T cells and ultimately suppressing colorectal cancer progression (21). Additionally, the metabolite butyrate produced by *Roseburia* induces M2-type TAM polarization, thereby promoting lung cancer metastasis (22).

Both the TM and the GM have been shown to have significant regulatory effects and therapeutic potential in TC. The TM of TC constitutes a core component of the TME and can interact directly with TME components such as tumor cells, immune cells, and tumor-associated fibroblasts, thereby participating in the remodeling of the local microenvironment of TC. Recently, several studies have sparked widespread interest in the TM in TC using advanced high-throughput sequencing technologies (23). However, further cellular and animal experiments are needed to comprehensively elucidate the interactions between TMs and TCs. Therefore, investigating the diagnostic roles and regulatory mechanisms of the microbiome in TC represents an exciting and rapidly evolving area of research in cancer biology and therapeutic development. This review systematically outlines the role of the TM in cancer progression, summarizes the alterations in the TM and TME in TC and their impact on TC progression, and further explores the potential mechanisms of the TM and TME in TC treatment, aiming to provide new insights for adjuvant therapeutic strategies in TC.

## 2 Methods

This study is a narrative review. A systematic search was conducted in major domestic and international databases, including PubMed, Web of Science, and Google Scholar. Literature was searched from the inception of each database to June 2026. The search strategy combined subject headings with free-text terms. Associated keywords were selected based on the core research themes, including “thyroid carcinoma”, “microbiome”, “immune microenvironment”, “progression”, and “cancer immunotherapy”. Clinical trials were searched on ClinicalTrials (https://clinicaltrials.gov/). Additionally, a manual search of the references in the included studies and reviews in related fields was conducted to identify any omitted literature.

The inclusion criteria were as follows: (1) formally published English literature whose research topics are closely related to the content of this review; (2) article types including clinical trials (human), research articles (human, animals, and *in vitro* experiments), meta-analyses, and letters; and (3) the literature must be complete, and the full text must be available.

The exclusion criteria were as follows: (1) conference abstracts, case reports, and noncore gray literature; (2) duplicated publications or literature with highly overlapping content; and (3) literature with obvious flaws in study design or missing key information.

## 3 Immune microenvironment in TC

**Figure 1:**
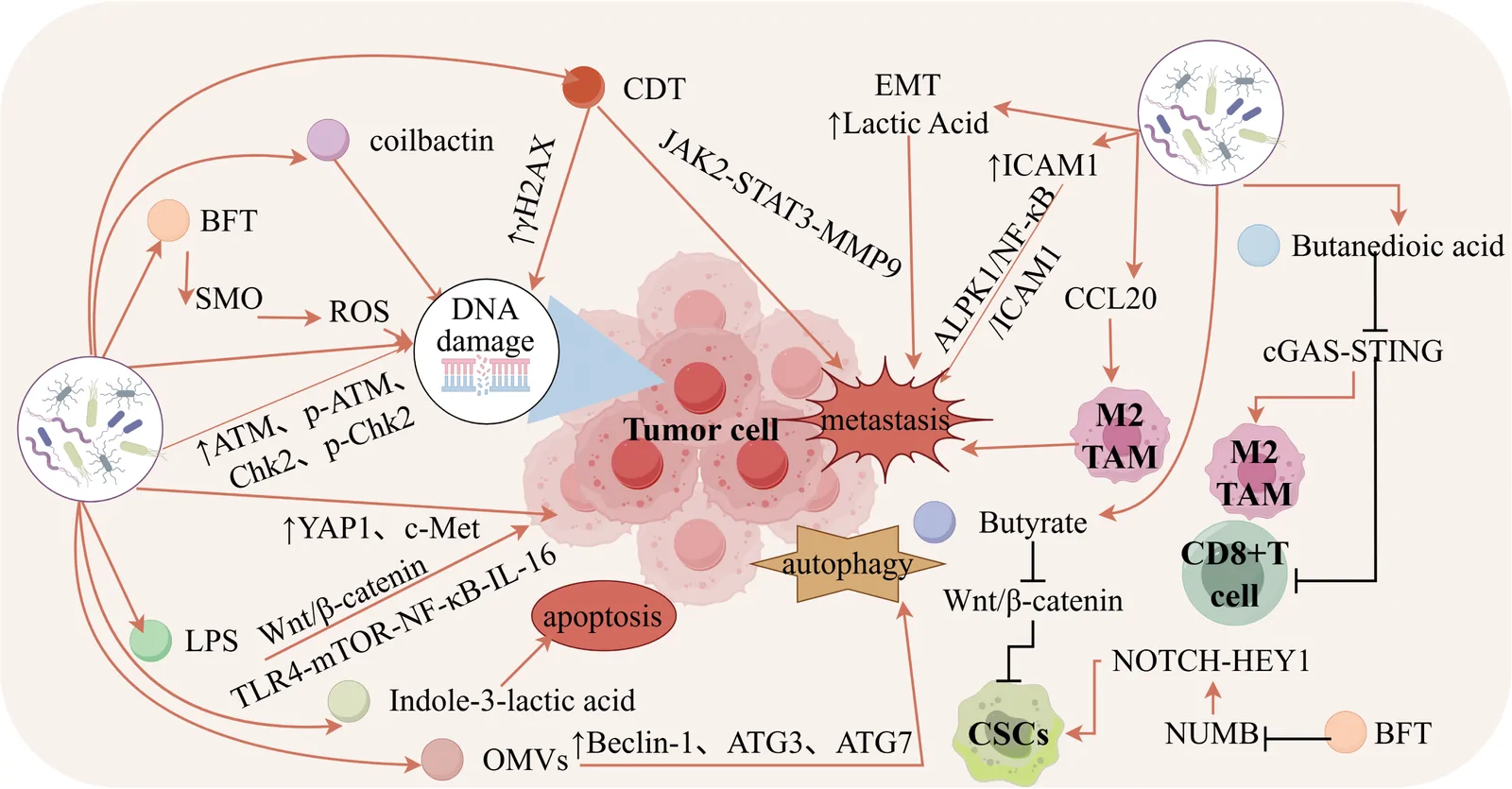
Immune microenvironment in TC. The TME in TC includes multiple cellular components, including cancer cells, CSCs, B cells, M1/M2-TAMs, T cells, NK cells, endothelial cells, and so on. Upregulated STAT6, DPP4, CAPN8, and HS3ST3A1 can either suppress the activity of CD8^+^T cells or NK cells, thus participating in the development of the immunosuppressive phenotype of the TME. (Created by Figdraw, ID: SISYR41e7e).

A growing body of evidence indicates that the TME affects the biological behavior of TC via distinct immune states. Multiple interrelated components of the immune system are involved in tumor invasion and are closely associated with TC metastasis. On the basis of an analysis of data from the TCGA database, Liang et al. suggested that in TC, the infiltration of M2-TAMs, resting mast cells, and DCs is significantly increased, whereas the infiltration of CD8-positive cells, M1-TAMs, activated mast cells, and eosinophils is reduced (24). Similarly, on the basis of data from the TCGA database, Xie et al. also suggested that PTC has increased infiltration of M2-TAMs, Tregs, monocytes, neutrophils, DCs, mast cells, and M0-TAMs, which promote tumor progression, whereas M1-TAMs, CD8^+^ T cells, B cells, NK cells, and follicular helper T cells have antitumor effects. During the development and progression of PTC, immune cells become activated, and the abundance and proportion of protumor immune cells significantly increase, indicating intensified immune evasion (25). The TME in TCs is illustrated in **Figure 1**.

### 3.1 Characteristics of T lymphocytes in terms of TC

Hou et al. performed single-cell RNA sequencing (scRNA-Seq) of human thyroid cancer tissues. They suggested that compared with PTC tissues, MTC tissues contain a lower proportion of immune cells (including T cells, B cells, myeloid cells, and, notably, CD45^+^ cells) but a greater proportion of basal cells. Therefore, MTC is believed to have an immunosuppressive microenvironment (26). Han et al. compared the differences in immune environments between PTC and ATC using scRNA-Seq analysis of human tumor tissues. The results revealed that cytotoxic cells (including CD8^+^ effector T cells and NK cells) are significantly more abundant in PTC samples than in ATC samples (27). Wang et al. analyzed human cancer datasets from the TCGA database. Compared with those in normal samples, the proportions of CD8^+^ T cells and Tregs are greater in PTC samples (28). In contrast, compared with ATCs, PTCs are more common (27). Through human samples, Zhang et al. demonstrated that CD8+ T cells in ATCs display severe metabolic dysfunction and an exhausted phenotype, whereas CD8+ T cells in PTCs exhibit relatively mild exhaustion (29).

Several biomarkers promote the progression of TC by activating oncogenic pathways and regulating the immunosuppressive microenvironment (30, 31). For example, Jing et al. performed bioinformatics analysis using datasets from the TCGA and GEO databases and confirmed that DPP4 is highly expressed in PTC and can induce CD8^+^ T-cell exhaustion and activate the IL13-IL13RA2 axis, facilitating the formation of an immunosuppressive TME (32). Furthermore, we explored how drugs influence immune responses through multiple pathways to exert therapeutic effects on TC. For example, curcumin has been shown to enhance CD8^+^ T-cell function in ATCs and augment antitumor immune responses by inhibiting the AKT/mTORC1/STAT3/PD-L1 signaling axis (33). In summary, modulating immune responses and targeting specific molecules, such as STRA6 and DPP4, can regulate the TME, providing new insights and potential approaches for immunotherapy in TC treatment.

### 3.2 Dendritic cells in the TC microenvironment

DCs are considered the most potent professional antigen-presenting cells (34) and key regulators of antitumor immunity (35). DCs can promote the activation of potent antitumor T cells and immune responses through various mechanisms, thereby influencing immune tolerance and cancer progression (36). In recent years, the role of DCs in cancer development has also been confirmed. DC-specific expression of the deoxyribonuclease DNASE1L3 can increase CD8^+^ T-cell infiltration, reduce TME exhaustion, significantly slow tumor growth, and increase anti-PD-L1 responses (37).

Existing studies have also demonstrated the critical role of DCs in treating TC. For instance, Wang et al. performed scRNA-Seq analysis using human samples (including 18 adjacent tissue samples and nonprogressive PTC and progressive PTC samples). They demonstrated that progressive PTC has high infiltration of lysosomal-associated membrane protein 3 (LAMP3^+^) dendritic cells (DCs), which are associated with advanced T stage and poor prognosis (30). Another study through the TCGA and GEO databases revealed that annexin A1 interacts with the formyl peptide receptor type 1 receptor in DCs, which can regulate the immune microenvironment in TC. Inhibiting this interaction reduces DC activity, ultimately suppressing the occurrence and development of TC (38). Therefore, mediating DC activity is believed to be a potential immunotherapy strategy against TC.

### 3.3 TAMs in the TC microenvironment

TAMs are among the major components of the TME and are closely associated with malignant progression and tumor metastasis (39). Previous studies have suggested increased infiltration of M2-TAMs and decreased infiltration of M1-TAMs in TC (24, 25). Lv et al. established a coculture system using TC cells and M2-TAMs. Ex vitro experiments have suggested that M2-TAMs can promote the dedifferentiation, proliferation, migration, and invasion of TC cells by activating Wnt/β-catenin signaling (40). In turn, human monocytes undergo “M2” polarization when cocultured with senescent thyrocytes or TC cells through the activation of PGE2 secretion, thus increasing tumor invasiveness (41). The interaction between TAMs and TCs in the TME plays an essential role in TC development. Future investigations targeting M2-TAM polarization could offer new strategies for the immunotherapy of TC.

### 3.4 CAFs and mast cells in the TC microenvironment

CAFs are a major component of the TME and play key roles in regulating tumor progression and immune escape. CAFs participate in the regulation of malignant tumor behaviors through the release of exosomes. Exosomal CREB1 derived from CAFs can promote TC cell proliferation and immune escape by positively regulating CCL20 expression (42). Additionally, *in vitro* studies have shown that CD36 expression in CAFs significantly enhances the proliferation, migration, and invasion abilities of PTC cells while inhibiting their apoptosis (43). Therefore, we speculate that targeted inhibition of CAF proliferation may become a novel approach to block TC progression and enhance immune activity.

Mast cells, as a type of innate immune cell, are often distributed in tumors. They can release presynthesized soluble mediators and suppress the body’s immune response to tumors. Compared with that in adjacent normal tissues, mast cell infiltration in TC tissues is significantly greater (23–25). *In vitro* experiments have confirmed that mast cells exert protumor effects on TC through the expression of galectin-9 to inhibit the antitumor function of CD8^+^ T cells. They also promote disease progression through the release of various cytokines and alterations in tumor stromal structure (44). Therefore, we hypothesize that intervening in mast cell proliferation may restore the antitumor immune activity of CD8^+^ T cells, thereby enhancing the efficacy of immunotherapy for TC.

## 4 TM and cancer progression

**Figure 2:**
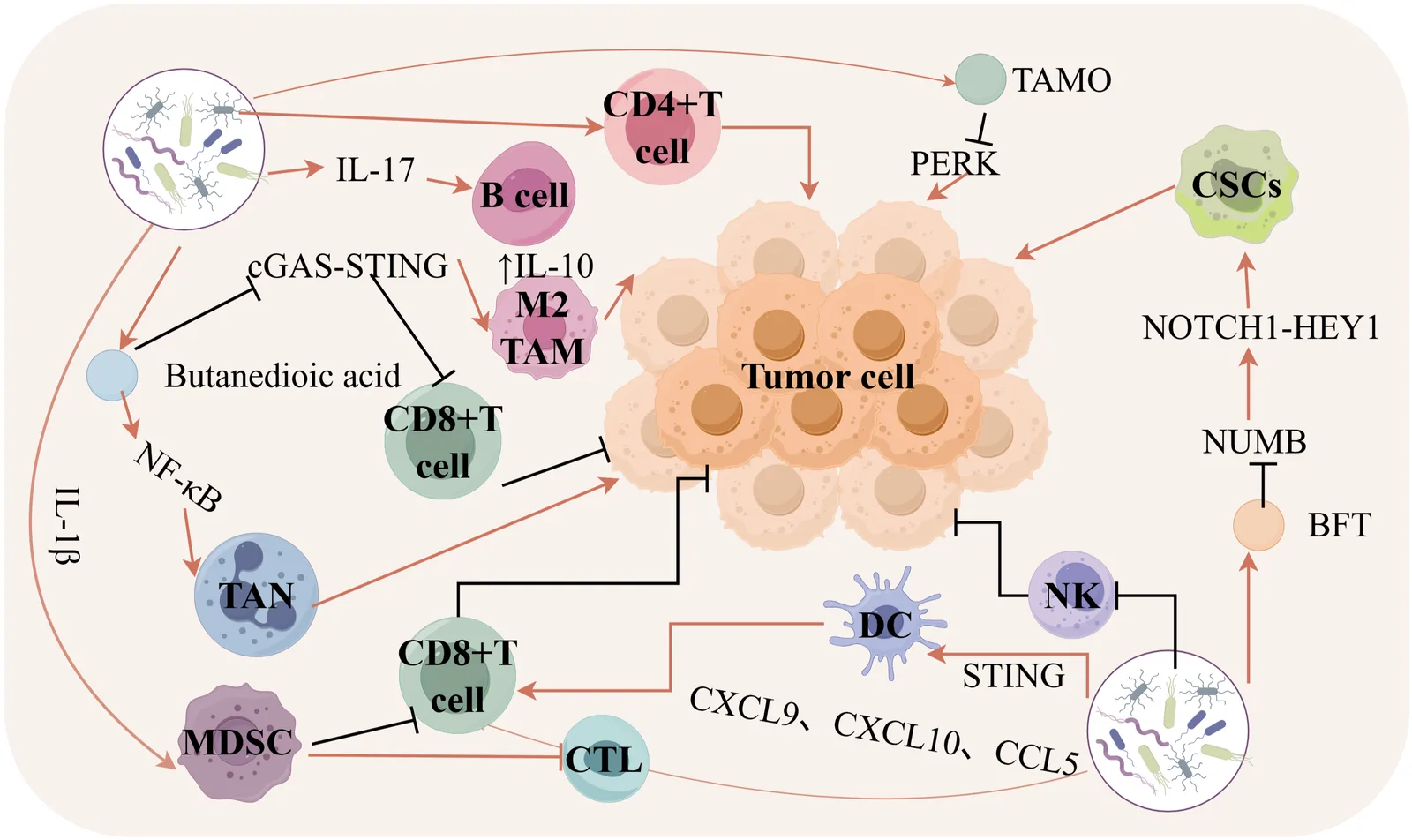
Effects of the TM on malignant behavior and TME. The TM exerts regulatory effects, such as DNA damage, metastasis, apoptosis, autophagy, proliferation, and TME of tumors. BFT, *Bacteroides fragilis* toxin; CDT, cytolethal distending toxin; CSCs, cancer stem cells; EMT, epithelial–mesenchymal transition; LPS, lipopolysaccharides; OMVs, outer membrane vesicles; ROS, reactive oxygen species; SMO, spermine oxidase; TAM, tumor-associated macrophages; TM, tumor microbiome; TME, tumor microenvironment; γH2AX, histone H2AX. (Created by Figdraw, ID: SISYR41e7e).

Potential sources of the TM include infiltration from the oral cavity, gut, and adjacent tissues into the tumor site. TMs play a crucial role in cancer progression by regulating various malignant tumor phenotypes, including DNA damage (45–52), cell proliferation and growth (53–59), metastasis (60–63), and the stemness of tumor cells (64–67). Meanwhile, TMs have significant effects on the TME and therapeutic sensitivity (**Figure 2**). Notably, the colonization patterns and functional regulation of the intratumoral microbiome significantly differ across different cancer types. Influenced by the organ microenvironment, endocrine properties, and local immune context, the mechanisms underlying microbiome–tumor interactions vary markedly among different tumors.

### 4.1 Effect of the TM on cancer proliferation and growth

The rapid proliferation and growth of tumor cells are key factors leading to the rapid development of cancer. Recent studies have shown that TM can influence the proliferation and growth of tumor cells, with some TMs directly promoting tumor cell growth. For example, *in vitro* experiments have confirmed that *F. nucleatum* can accelerate cancer cell growth by increasing the levels of protumor proteins such as YAP1 and c-Met (53). This bacterium can also induce PC cells to secrete cytokines, enhancing PC cell proliferation (54); Sha et al. (55) demonstrated that lipopolysaccharide (LPS), produced by *Escherichia-Shigella* and unclassified *f:Enterobacteriaceae*, can activate the TLR4-mTOR-NF-κB-IL-6 axis and drive NSCLC cell proliferation. Wild-type mice exhibited significant liver inflammation, fibrosis, and dysplasia when they received fecal microbiota transplantation (FMT) from patients. The abundance of *Klebsiella pneumoniae*, a gut pathogen, was enriched in the liver tissues of mice following FMT. Mechanistically, *the K. pneumoniae* surface protein PBP1B interacts with and activates TLR4 in HCC cells, thereby promoting HCC cell proliferation and the activation of oncogenic signaling. This bacterium can also disrupt the intestinal barrier (56). Deng et al. established a CRC mouse model and verified that *F. mortiferum* and its metabolite 5-aminonorvaline can activate the Wnt/β-catenin pathway to promote tumor progression and significantly increase CRC cell proliferation (57).

Additionally, studies have revealed that TM and its metabolites inhibit the proliferation of tumor cells through various pathways. For example, *in vitro* experiments have confirmed that *Lactobacillus johnsonii* (*L. johnsonii*) suppresses PTC cell proliferation by inhibiting the Wnt/β-catenin pathway (58). Yang et al. isolated Lactobacillus paracasei (L. paracasei) ZJUZ2–3 from GC patients and administered it to mice, confirming that this strain secretes indole-3-acetic acid, which inhibits GC cell survival by suppressing the NF-κB signaling pathway (59).

On this basis, TM has been confirmed to directly regulate the proliferation and growth of tumor cells through multiple pathways, bidirectionally influencing cancer progression. This may be related to the type of microorganism (probiotic or pathogenic) and the role of its metabolites in tumor proliferation.

### 4.2 TM and cancer metastasis

Cancer metastasis is the leading cause of death in cancer patients. Recent research has shown that TM and its metabolites can regulate the metastatic process through various mechanisms.

TM and its metabolites can directly promote cancer metastasis by regulating metabolism and immune suppression. For example, through human samples, Gu et al. demonstrated that *E. coli* can promote CRC liver metastasis by enhancing intratumoral glycolysis and lactate production (60). He et al. performed animal experiments and suggested that *C. jejuni*-produced CDT promotes liver or lung metastasis in CRC mice through the JAK2-STAT3-MMP9 signaling pathway, with the prometastatic effect primarily dependent on the CDT subunit cdtB (61). Xu et al. demonstrated that patients with CRC had an increased abundance of *F. nucleatum* in feces and tumors. Further cellular and animal studies suggest that *F. nucleatum* promotes TAM infiltration via CCL20 activation while inducing M2-TAM polarization, thereby enhancing CRC lung metastasis (62). *In vitro* experiments have confirmed that this bacterium also enhances adhesion between CRC cells and endothelial cells, induces ICAM1 expression, and subsequently activates the ALPK1/NF-κB/ICAM1 axis to promote distant CRC metastasis (63). Moreover, Ma et al. demonstrated that *Roseburia*-derived butyrate promotes LC metastasis by inhibiting HDAC expression, upregulating H19 expression in tumor cells, and inducing M2-TAM polarization (22). On this basis, targeting TM to inhibit its cancer-promoting metastatic functions could advance tumor therapy development.

### 4.3 TM and cancer immune microenvironment

**Figure 3:**
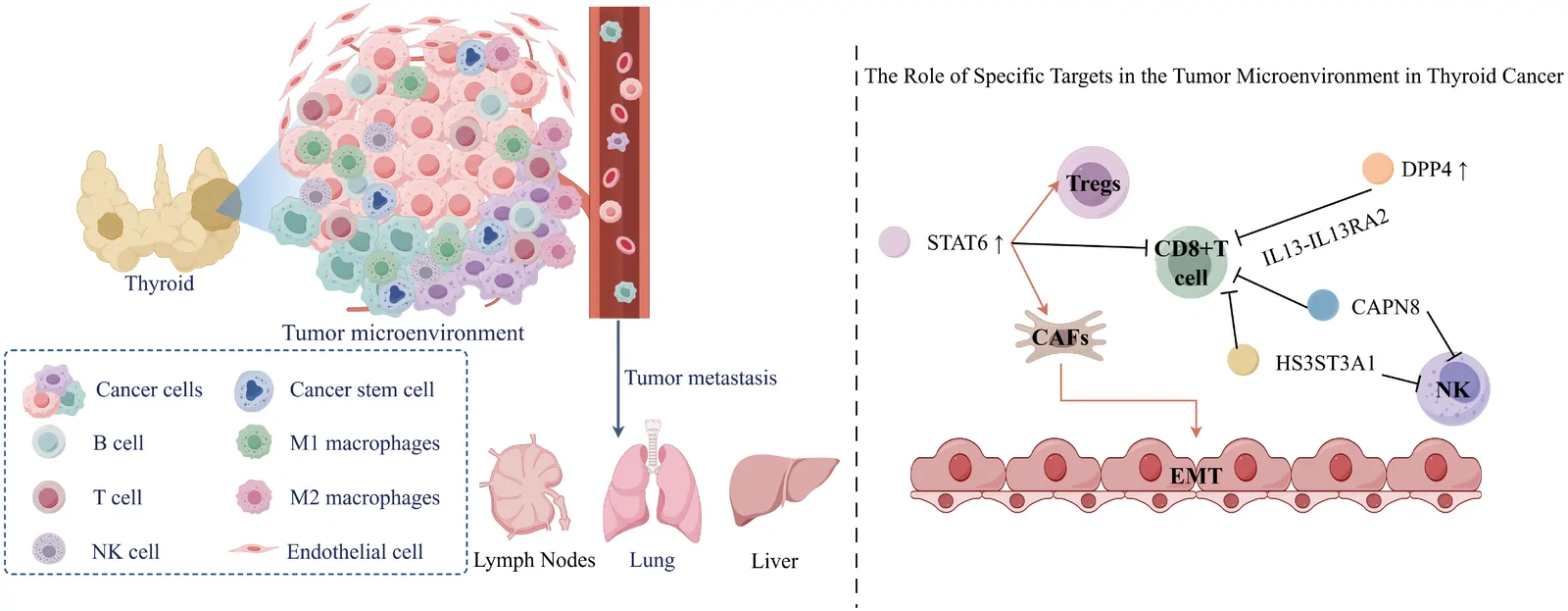
Mechanisms of the TM in the immune TME. TM exerts regulatory roles in the immune TME by multiple pathways: such as IL-1β, cGAS-STING, PERK, NUMB-NOTCH1-HEY1, IL-17, NF-κB, CXCL9, CXCL10, and CCL5. BFT, *Bacteroides fragilis* toxin; CSCs, cancer stem cells; CTL, cytotoxic T lymphocyte; DC, dendritic cells; IL-10, interleukin-10; IL-1β, interleukin-1β; MDSCs, myeloid-derived suppressor cells; NK, natural killer cells; TAM, tumor-associated macrophages; TAMO, trimethylamine N-oxide; TM, tumor microbiome; TME, tumor microenvironment. (Created by Figdraw, ID: SISYR41e7e).

TMs play a crucial role in cancer progression by regulating the tumor immune microenvironment. The TME contains a large number of immune cells, including CD4+ T cells, CD8+ T cells, natural killer (NK) cells, regulatory T cells (Tregs), TAMs, DCs, B cells, and neutrophils. These cells significantly influence tumor proliferation and development, with the main mechanisms illustrated in **Figure 3**.

TMs can mediate tumor immunity through various pathways to influence tumor progression. For example, Jiang et al. confirmed that fecal transplantation from CRC patients into germ-free mice allows succinate, a metabolite produced by *F. nucleatum*, to inhibit the cGAS-STING pathway, thereby suppressing the transport of CD8^+^ T cells *in vivo* and converting TAMs into immunosuppressive cells, exacerbating colorectal cancer (68). Zhang et al. (69) determined the abundance of the intratumoral fecal microbiome transplantationin human gastric cancer tissues and reported that *F. nucleatum* was significantly enriched in tumor tissues with lymph node metastasis and associated with poor prognosis. Animal studies have suggested that *F. nucleatum* infection induces chronic gastritis, promotes gastric mucosa dysplasia, activates the NF-κB signaling pathway, promotes the recruitment of tumor-associated neutrophils, and facilitates immune evasion in gastric cancer. Tan et al. (70) established a PC mouse model and verified that *P. gingivalis* promotes the progression of pancreatic cancer by increasing the secretion of neutrophil chemokines and neutrophil elastase.

It has been confirmed that targeting the TM can inhibit tumors by affecting the immunosuppressive TME. TM can also suppress tumor progression by regulating immune responses. In mice bearing breast cancer cells, combining *Megasphaera* with immune checkpoint inhibitor anti-programmed cell death-1 (PD-1) therapy increases the number of CD4+ T cells and dendritic cells (DCs). The combination of these two approaches can inhibit tumor growth by influencing the TME, thereby achieving therapeutic effects (71). Additionally, colonizing *Streptococcus* in mice with esophageal squamous cell carcinoma promoted the infiltration of CD8+ T cells and enhanced the efficacy of anti-PD-1 immunotherapy, revealing its potential role in cancer treatment (72). Furthermore, metabolites produced by TMs also play a role in tumor therapy. For instance, the metabolite butyrate, produced by *Butyrivibrio, Clostridium*, and *Faecalibacterium*, can increase the sensitivity of endometrial cancer patients to progesterone and induce ferroptosis through CISD1 downregulation, thereby reducing progesterone resistance (73). Using three different homologous mouse models of lung cancer, Liu et al. demonstrated that *A. sydowii* can promote lung tumor progression by mediating IL-1β-dependent expansion and activation of myeloid-derived suppressor cells, leading to reduced cytotoxic T lymphocyte (CTL) activity and inhibition of PD-1^+^ CD8^+^ T-cell accumulation (74). In summary, TM can exert immunosuppressive effects through targeted therapy on tumor development and reduce drug resistance via its metabolites, highlighting the potential clinical value of TM in treating tumors through its ability to regulate the tumor immune microenvironment.

### 4.4 TM and cancer apoptosis, autophagy and chemoresistance

TMs play important roles in multiple stages of tumor development. By specifically targeting the TM and regulating its composition or function, it is expected to become a new adjuvant therapeutic approach to enhance the efficacy of existing therapies and inhibit tumor progression. TM can promote cancer apoptosis. For example, Sugimura et al. demonstrated that *Lactobacillus gallinarum* can increase the abundance of probiotics in a CRC mouse model and reduce intestinal pathogens and that its secreted indole-3-lactic acid can promote CRC apoptosis, thereby inhibiting tumorigenesis (75). *In vitro* experiments have confirmed that *Streptococcus thermophilus* secretes β-galactosidase, which can induce CRC cell cycle arrest in the G0/1 phase and subsequently trigger apoptosis (76).

Studies have confirmed that TM can induce cancer cell autophagy. Chen et al. used a mouse model of lung cancer and reported that *F. nucleatum*-secreted outer membrane vesicles (Fn OMVs) increase the mRNA levels of Beclin-1, ATG3, and ATG7. Fn OMVs activate intracellular autophagy pathways and induce the expression of epithelial–mesenchymal transition (EMT)-related proteins, thus promoting lung metastasis in tumor-bearing mice (77). On the basis of human samples, Yu et al. demonstrated that *F. nucleatum* was more abundant in CRC tissues from patients with recurrence after chemotherapy than in those from nonrecurrent patients. High abundance of *F. nucleatum* in CRC patients is associated with shorter recurrence-free survival and five-year recurrence survival (78).

A relatively high abundance of *F. nucleatum* has been detected in patients with *cancer with* chemoresistance (78, 82). Yu et al. further confirmed that when CRC cells were cocultured with *F. nucleatum*, the autophagy pathway was activated, thus inducing resistance to oxaliplatin and 5-FU (78). Dong et al. established a mouse CRC model using azoxymethane (AOM)/dextran sodium sulfate (DSS). FMT enhanced radiation-mediated antitumor effects in this model and prevented adverse events. More *Roseburia intestinalis* accumulated in the gastrointestinal tract after FMT. Oral gavage of *Roseburia intestinalis* and butyrate synergistically increased radiation sensitivity but alleviated intestinal toxicity by promoting CRC cell autophagy (79). Furthermore, TM can induce chemotherapy resistance in cancer cells. *Roseburia intestinalis* enhances CRC cell chemosensitivity through the butyrate/OR51E1/RALB axis (79). Ding et al. established a CRC mouse model and verified that *B. fragilis* can activate the Notch1 signaling pathway to induce EMT and inhibit apoptosis, thereby promoting chemoresistance in CRC cells and xenografts (80). *In vitro* experiments have confirmed that *E. coli* produces colibactin, which induces lipid droplet accumulation, increases ROS levels, and promotes chemoresistance (81). Cellular and animal experiments have confirmed that *F. nucleatum* upregulates BIR3 expression via the TLR4/NF-κB pathway, thus promoting CRC cell resistance to 5-Fu (82).

In summary, TM and its metabolites can inhibit tumors by suppressing cancer apoptosis, but they can also promote cancer cell autophagy and increase chemoresistance, thereby advancing tumor progression.

## 5 The alterations and roles of TM in TC development

**Figure 4:**
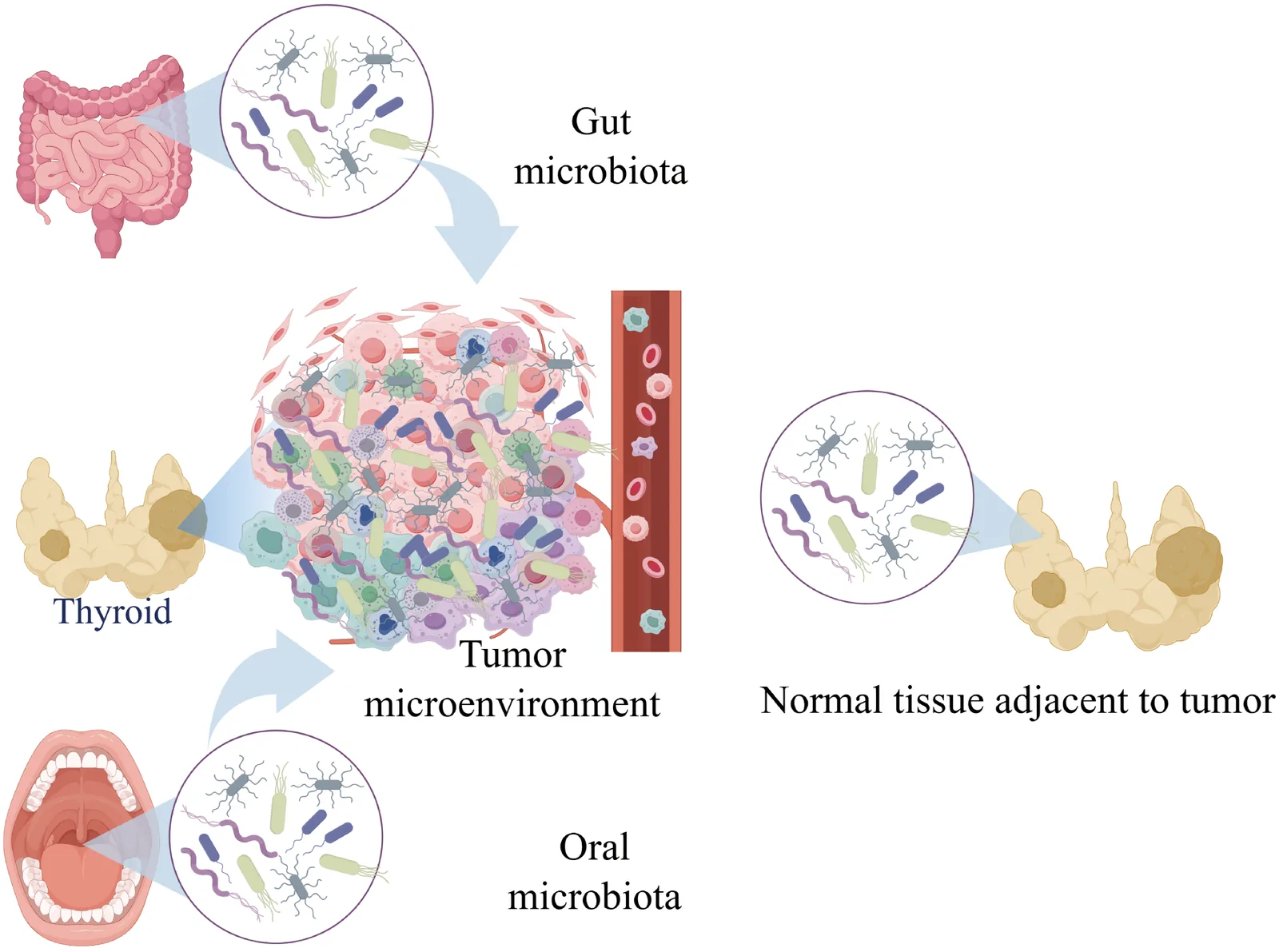
The potential origins of the intratumor. The microorganisms in the thyroid tissue of the TC primarily originate from the oral cavity, intestines, and adjacent normal tissues of the tumor. (Created by Figdraw, ID: SISYR41e7e).

**Figure 5:**
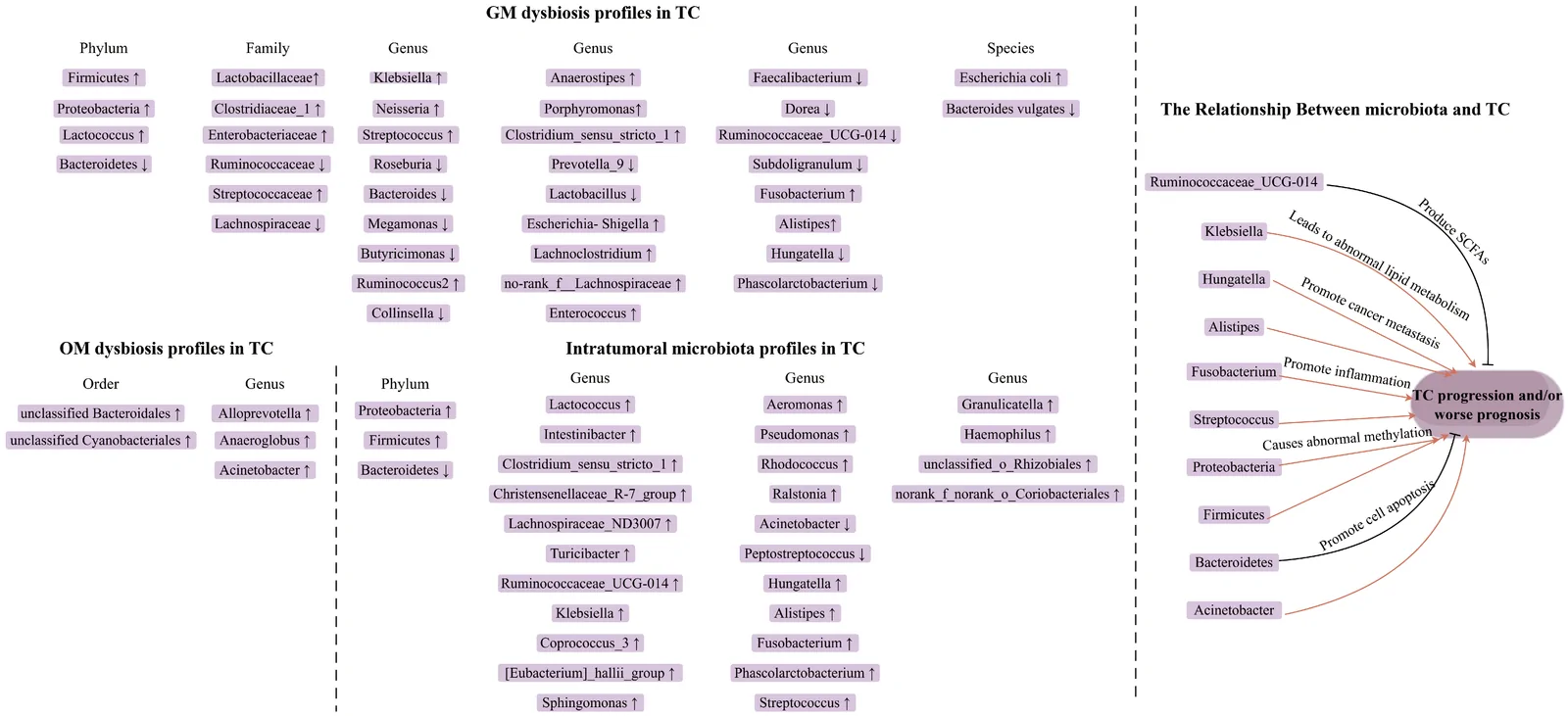
GM and TM profiles in TC. The GM, OM and TM characteristics and their interaction mechanisms associated with TC. The GM dysbiosis profile involves four taxonomic levels (phylum, family, genus, and species) with significant abundance abnormalities. The OM dysbiosis profile is centered on the order and genus taxonomic levels. The TM profile exhibited abnormal abundance characteristics of microorganisms colonizing tumor tissues. In addition, the right side of the figure shows the interactions between microorganisms and TC. (Created by Figdraw, ID: SISYR41e7e).

The TM in the TC typically originates from the gut (83), oral cavity (23), and adjacent normal tissues (**Figure 4**). In recent years, studies have reported significant alterations in the GM and TM of patients with TC (83, 84). For instance, compared with healthy controls, TC patients exhibit increased abundances of *Firmicutes*, *Proteobacteria*, and *Lactococcus* at the phylum level, while *the* abundance of Bacteroidetes is reduced (85, 86). The combination of 8 metabolites and 5 genera was highly effective at distinguishing TC patients from HCs (AUC = 0.97), suggesting that the altered GM has potential as a diagnostic biomarker (85). Notably, animal experiments have verified that thyroid dysfunctions that are induced by chemical or surgical methods cause significant changes in GM compositions (87). In turn, alterations of GM are supposed to affect TM (20) and thyroid functions (88). Those studies indicate the crucial crosstalk among GM, TM, and TC (**Figure 5**).

### 5.1 TM characteristics in TC

An increasing number of studies have focused on exploring the characteristics of the TM in patients with TC. Dai et al. collected tumor tissues and adjacent peritumor tissues from 30 patients with TC and conducted 16S rRNA gene sequencing. They reported significant differences in microbial composition and diversity between tumor tissues and adjacent tissues. Specifically, *Sphingomonas*, *Comamonas*, *Acinetobacter*, *Pseudomonas*, *Microvirgula*, and *Soonwooa* composed the core microbiome of the thyroid. The abundances of *Sphingomonas* and *Aeromonas* markedly increased in tumor tissues, whereas the abundances of *Comamonas*, *Acinetobacter*, and *Peptostreptococcus* significantly increased in adjacent tissues (83). In addition, Qiu et al. reported that the abundance of *Delftialacustiris* is significantly lower (P = 0.0057) in PTC tissues than in normal thyroid tissues (89).

Current research on the TM of TC focuses primarily on PTC, the most prevalent subtype of TC. The incidence of PTC differs significantly across sexes, with a higher incidence in female patients, while male patients are often diagnosed at an advanced stage of the disease (90). Investigations based on the TCGA database revealed that 88 species of fungi and archaea are significantly dysregulated in females, whereas only 11 species of fungi and archaea are significantly dysregulated in males (91). The microbiome composition in TC also varies between males and females (92). Wang et al. suggested that the distribution of the same microorganism also differs by sex. *Synechococcus* sp. CC9311 is enriched in normal male samples but is more prevalent in tumor tissues in females (93). Additionally, Yuan et al. collected tumor samples from 80 patients with PTC and analyzed the TM using 16S RNA sequencing. Compared with male patients, male patients exhibited the greatest number of uniquely dysregulated pathways in tumor suppression-related groups, whereas female patients exhibited the most uniquely dysregulated pathways in DNA checkpoints and damage-related groups. Furthermore, compared with patients with earlier-stage disease (T1 or T2), patients with advanced-stage disease (T3 or T4) have significantly greater tumor bacterial α diversity (94). These studies suggest that alterations in the tumor microbiome are closely related to TC progression.

### 5.2 Effects of the TM on the malignant behaviors of TC cells

**Figure 6:**
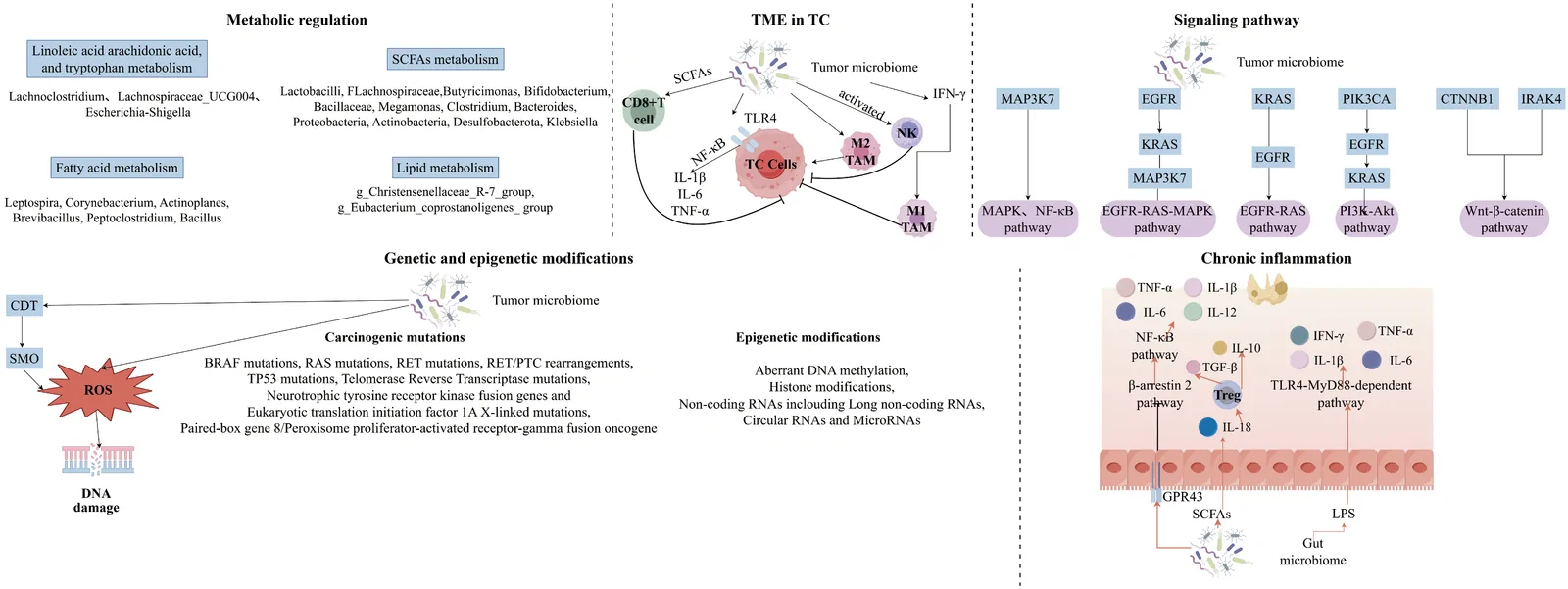
Potential roles and mechanisms of TM in TC development. TMs mediate multiple regulatory mechanisms underlying the occurrence and progression of TC, covering metabolic regulation, immune regulation, activation of signaling pathways, genetic and epigenetic modifications, and chronic inflammation. TC, thyroid carcinoma; CDT, cytolethal distending toxin; NK, natural killer cells; ROS, reactive oxygen species; SCFAs, short-chain fatty acids; SMO, spermine oxidase; TAM, tumor-associated macrophages; TM, tumor microbiome. (Created by Figdraw, ID: SISYR41e7e).

Microorganisms may play pivotal roles in modulating immune cell expression and regulating immune and cancer-related pathways to impede cancer progression (**Figure 6**). On the basis of 55 tumor tissues from PTC patients and 16S rRNA sequencing analysis, TC-enriched bacteria and various carcinogenic pathway-related genes were found to be positively correlated. Cellular and animal experiments have suggested that *L. johnsonii* can inhibit the proliferation, migration, and invasion of PTC cells by suppressing the Wnt/β-catenin signaling pathway, suggesting that the development and progression of thyroid tumors are closely related to intratumoral microbial dysbiosis (58).

TMs are typically found in organs with mucosal surfaces, such as the colon, pancreas, cervix, and lungs (95). These organs have cavities exposed to the external environment. During tumorigenesis, the mucosal barrier may be disrupted, creating opportunities for microorganisms on the mucosal surface to invade the tumor (96). Microbiome-produced metabolites, such as butyrate, propionate, and acetate, have potent effects on regulating immune responses and inflammation (97). Butyrate is a short-chain fatty acid derived from the fermentation of carbohydrates by the microbiome. It has been shown to slow tumor growth by influencing immune cells, tumor cells, and healthy gut cells (22, 98). Research has revealed a notable decline in the abundance of butyrate-producing bacteria, including *Blautia*, *Lachnoclostridium*, *Eisenbergiella*, *Flavonifractor*, and *Hungatella*, in PTC (99). *In vitro* experiments have suggested that TC cells exhibited enhanced sensitivity and DNA damage to radiotherapy following sodium butyrate treatment (100). Therefore, targeting butyrate by supplementation of sodium butyrate and/or in combination with components that stimulate the production of butyrate (e.g., dietary fiber, omega-3 fatty acids) might be a potential therapeutic strategy for TC (101).

## 6 Challenges and future perspectives

While preclinical and clinical investigations have revealed the essential roles of TM in the immune microenvironment and therapeutic responses of cancers, such as HCC, lung cancer, CRC, and ESCC, current research on TC is mostly limited to comparative analyses of the microbial composition between TC patients and healthy people. Studies based on large-scale clinical samples and diverse experimental verification methods are lacking, particularly in mechanistic investigations. Existing research on TM in TC focuses heavily on the PTC subtype, with insufficient systematic data on TM composition, microbe–immune crosstalk, and regulatory mechanisms in other TC subtypes. Moreover, most studies have been performed on the basis of single microbial sources, and investigations of the interplay between microbes across different ecological niches are lacking. Thus, this review has limitations in performing cross-subtype comparisons or establishing a universal microbe–immune interaction framework covering all TC subtypes—another major limitation of this review.

To overcome these limitations, standardized sample collection procedures, as well as optimized sequencing and bioinformatics pipelines, should be used, thus reducing clinical bias and improving the reproducibility and comparability of the results. Future research is urgently needed to explore the association between TM multiomics and the tumor immune microenvironment in FTC, MTC, and ATC. These findings clarify microbial heterogeneity across TC subtypes and facilitate the establishment of integrated subtype-specific models for microbiome–immune interaction analysis. Although FMT shows promising therapeutic potential for TC, its clinical application is associated with non-negligible safety risks, including pathogen transmission (due to inadequate donor screening), short-term gastrointestinal adverse events (abdominal distension, diarrhea, abdominal pain), and unclarified long-term risks (e.g., metabolic disorders and immune imbalance), and large-scale long-term follow-up verification is lacking.

Microbial communities play crucial roles in the initiation and progression of TC. However, several inconsistent findings merit future exploration. Chang et al. compared the differences in abundance of the microbiome in thyroid tissues and oral/fecal samples. They reported that these three sources had similar microbial diversity patterns, indicating potential microbial translocation or systemic interactions in the body (102). Compared with matched peritumor tissue samples, TC tumor tissue samples exhibit lower richness and diversity of the microbiome (83). Fernandes et al. (103) suggested that the abundance of *Faecalibacterium prausnitzii* was lower in TC patients than in volunteers. However, Jiang et al. (104) reported that *Faecalibacterium* was more abundant in healthy individuals than in DTC patients. These inconsistencies may result from variations in sample processing and experimental detection methods across studies.

## 7 Conclusion

Thyroid microbiome in TC exhibits prominent alterations in terms of richness and diversity compared with adjacent thyroid tissues and normal thyroid tissues, serving as potential diagnostic biomarkers. TM and metabolites impact the malignant phenotypes of human cancers and regulate immunotherapeutic efficacy by remodeling the immune TME in a context-dependent manner. Notably, alterations of thyroid microbiome and metabolites (e.g., cholesterol and butyrate) are associated with TC progression and heterogeneous activation of immune cells in the TME of PTs, MTCs, and ATCs. Emerging strategies include dietary modulation, FMT, probiotics, and rational combination therapies. In future research, longitudinal multi-omics analyses and prospective clinical validation should be employed to uncover causal mechanisms and establish reproducible biomarkers grounded in functional assessment. Targeting the interactions between the microbiome and immune microenvironment might be a potential strategy for optimizing TC therapies in the clinic.

## Glossary

A.sydowii: Aspergillus sydowii
Akk: Akkermansia muciniphila
ATC: Anaplastic Thyroid Carcinoma
B.fragilis: *Bacteroides fragilis*
BC: Breast Carcinoma
BCa: Bladder Carcinoma
BFT: *Bacteroides fragilis* toxin
C.Jejuni: Campylobacter. Jejuni
CAFs: Cancer-associated fibroblasts
CCA: Cholangiocarcinoma
CDT: Cytolethal distending toxin
CRC: Colorectal Carcinoma
CSCs: Cancer stem cells
CTL: Cytotoxic T Lymphocyte
DC: Dendritic Cells
DSBs: DNA double-strand breaks
E.coli: *Escherichia coli*
EAC: Esophageal Adenocarcinoma
ECM: Extra Cellular Matrix
EMT: Epithelial–mesenchymal transition
ESCC: Esophageal Squamous Cell Carcinoma
FMT: Fecal microbiota transplantation
F.nucleatum: *Fusobacterium nucleatum*
FTC: Follicular Thyroid Carcinoma
GC: Gastric Carcinoma
GIST: Gastrointestinal Stromal Tumor
GM: Gut microbiome
HCC: Hepatocellular Carcinoma
L.johnsonii: Lactobacillus johnsonii
L.paracasei: Lactobacillus paracasei
LAMP3+: Lysosomal associated membrane protein 3
LLC: Lewis Lung Carcinoma
LPS: Lipopolysaccharides
LSCC: Lung Squamous Cell Carcinoma
LUAD: Lung Adenocarcinoma
MTC: Medullary Thyroid Carcinoma
NK: Natural killer cell
NSCLC: Non-Small Cell Lung Carcinoma
OSCC: Oral Squamous Cell Carcinoma
PC: Pancreatic Carcinoma
PD-1: Programmed death-1
PDAC: Pancreatic Ductal Adenocarcinoma
PD-L1: Programmed cell death ligand 1
PSA: Prostate Carcinoma
PTC: Papillary Thyroid Cancer
SMO: Spermine oxidase
TAM: Tumor-associated macrophages
TC: Thyroid Carcinoma
TM: Tumor microbiome
TME: Tumor Microenvironment
Tregs: Regulatory T cells
TSH: Thyroid stimulating hormone
γH2AX: Histone H2AX
